# Characterisation of Allergen Profiles in Commercial Egg Products to Inform Oral Immunotherapy Strategies in Egg Allergy

**DOI:** 10.1111/cea.70387

**Published:** 2026-07-02

**Authors:** Elecia B. Johnston, Shaymaviswanathan Karnaneedi, Eva Birosova, Paxton Loke, Adriana Chebar Lozinsky, Lydia Su‐Yin Wong, Agnes Sze‐Yin Leung, Elizabeth Huiwen Tham, Lynette Pei‐Chi Shek, Mimi L. K. Tang, Andreas L. Lopata

**Affiliations:** ^1^ Molecular Allergy Research Laboratory, College of Science and Engineering James Cook University Townsville Queensland Australia; ^2^ Australian Institute of Tropical Health and Medicine James Cook University Townsville Queensland Australia; ^3^ Centre for Food and Allergy Research Murdoch Children's Research Institute Melbourne Victoria Australia; ^4^ Allergy Immunology Murdoch Children's Research Institute Parkville Victoria Australia; ^5^ Department of Paediatrics The University of Melbourne Parkville Victoria Australia; ^6^ Monash Children's Hospital Clayton Victoria Australia; ^7^ Khoo Teck Puat‐National University Children's Medical Institute National University Health System Singapore Singapore; ^8^ Department of Paediatrics The Chinese University of Hong Kong Hong Kong Hong Kong; ^9^ Department of Paediatrics National University Singapore, Yong Loo Lin School of Medicine Singapore Singapore; ^10^ Department of Allergy and Immunology Royal Children's Hospital Parkville Victoria Australia; ^11^ Tropical Futures Institute James Cook University Singapore Singapore Singapore

**Keywords:** allergen diagnostic, desensitisation, egg allergens, egg allergy, egg white powder, food allergy, oral immunotherapy, ovomucoid, ovotransferrin, probiotic

## Abstract

- Egg white products show substantial variability in major and minor allergen content across commercial preparations.
- Allergen variability may influence oral immunotherapy safety; product interchangeability can lead to inconsistent allergen dosing outcomes.

To the Editor,

Hen's egg allergy is one of the most common food allergies in children, affecting usually 0.5%–2.5% globally, with a higher prevalence of 16.5% in Australian infants, reflecting geographic variation in food allergy prevalence [1, 2]. Oral immunotherapy is a potential treatment; however, no standardised products exist, and current approaches rely on “off‐the‐shelf” egg preparations [3, 4]. Accurate allergen dosing is critical, yet the composition of commercial egg products remains poorly characterised.

The protein‐rich egg white (EW) contains the major allergens ovomucoid (OVM, Gal d 1), ovalbumin (OVA, Gal d 2), ovotransferrin (OVT, Gal d 3), and lysozyme (Gal d 4). OVA is the most abundant allergen in EW, while OVM is immunodominant and heat‐stable, whereas other allergens vary in stability during processing [5]. These differences may alter allergen composition across products, as observed in other foods such as peanut [6, 7]. Such variability has important implications for the use of commercial egg products in immunotherapy. In this study, we aimed to quantify allergen content in commercial powdered and liquid EW products and assess variability across preparations to inform their suitability for immunotherapy.

Four powdered (P1–P4) and three liquid (L5–L7) EW products were analysed. Standardised protein extracts and their allergen content were analysed by sodium‐dodecyl‐sulfate‐polyacrylamide gel‐electrophoresis (SDS‐PAGE), mass spectrometry (MS), ELISA, and immunoblot. Tryptic digestion preceded MS for peptide identification and relative allergen quantification. Commercial ELISA allergen kits were used to quantify OVM, OVT, and lysozyme across the preparations (OVM and lysozyme from Eurofins; OVT from Abcam).

SDS‐PAGE revealed considerable variation in protein content, greater among powders than liquids, including a low molecular weight band absent in P1 and P3 (Online Repository: Figure E1, https://zenodo.org/records/19674183). MS confirmed the presence of the four major EW allergens (OVM, OVA, OVT, lysozyme) in all extracts (Figure 1A). These allergens accounted for 80%–85% of total proteins in the extracts. The relative abundance of OVM, OVA, and OVT were largely consistent across the powder and liquid preparations. However, lysozyme was present at much lower levels in P1 and P3 compared with other powder and liquid preparations.

**FIGURE 1:**
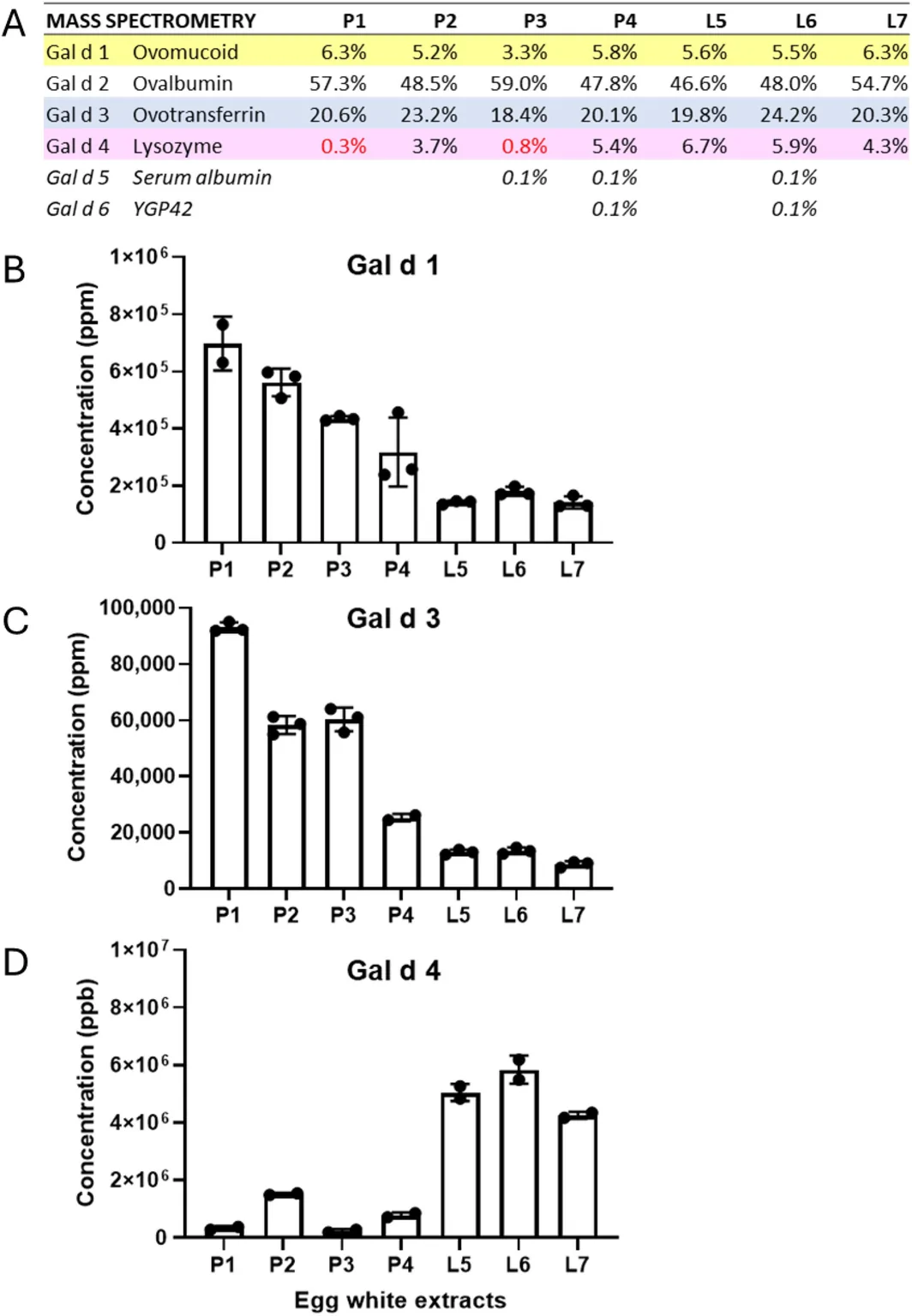
(A) Allergen abundance determined by mass spectrometry, calculated as percentage of total proteins. (B–D) Allergen quantification by ELISA. Concentration of Gal d 1 (OVM), Gal d 3 (OVT), and Gal d 4 (lysozyme), determined in four egg white powders (P1‐4) and 3 egg white liquids (L5‐7). Percent variation of OVM, OVT and lysozyme = 54%, 72%, 84% respectively for powders, and 21%, 35%, 27% for liquids.

ELISA measurements demonstrated variability in allergen concentrations between products (Figure 1B–D), with notable differences between powdered and liquid forms. Substantial variation was observed among the powdered products, whereas the concentration of allergens was comparatively consistent across liquid products (54%–84% variation across powders, and 21%–35% variation across liquids). OVM (Gal d 1) and OVT (Gal d 3) were highest in P1, with reduced levels in P2–P4 (OVM: 45%–80%; OVT: 27%–65%) and markedly lower concentrations in liquid products (OVM: 20%–25%; OVT: 9%–15% of P1), requiring ~1.5–2‐fold (powders) and up to 10‐fold (liquids) higher quantities to achieve comparable levels. In contrast, lysozyme (Gal d 4) was highest in liquid extracts, particularly L6, with L5 and L7 retaining 73%–86%, while powders contained substantially less (4%–26% of L6), necessitating ~1.2–1.4‐fold (liquids) and up to 25‐fold (powders) higher quantities to match L6 concentrations. OVM and OVT were less susceptible to the powder manufacturing process than lysozyme, resulting in a relative enrichment of these allergens within the powdered products. Conversely, the reduced lysozyme concentration in several of the powdered products, compared to other powdered and liquid products, suggests partial degradation of this allergen during the powder manufacturing process.

Immunoblotting was performed using sera from 13 egg‐allergic individuals and 10 controls (Online Repository: Table E1) to assess IgE binding. Multiple bands were visible in the immunoblots, confirming the presence of IgE binding proteins in the extracts (powdered products P1, P2 and fresh liquid EW L5) (Online Repository: Figure E2). There were four major IgE‐binding bands across the three protein extracts (~72, 50, 40, and 32 kDa), with bands being unique to egg‐allergic individuals (40 and 32 kDa) or showing increased intensity compared to negative control individuals (72 and 40 kDa). Patient IgE binding patterns were similar for the liquid EW (L5) and powdered EW (P1, P2), indicating sufficient concentration of allergens in the powders to elicit IgE‐allergen interaction.

Our findings revealed significant variability in allergen concentrations between commercially available egg products, as measured by both biochemical and antibody‐based tests. While MS revealed relatively consistent levels of OVM, OVA, and OVT across products, ELISA detected substantial differences in the concentrations of all four major allergens. This discrepancy likely arises from the different detection mechanisms: MS identifies allergen‐derived peptides regardless of protein structure, whereas ELISA detects whole proteins with intact epitopes recognised by antibodies. As a result, ELISA more accurately reflects clinically relevant allergen variability, highlighting the importance of immunoassay‐based methods in validating dosing for oral immunotherapy.

The variability detected by ELISA cannot be attributed to extraction artefacts, as overall protein recovery was consistent across preparations, as demonstrated by SDS‐PAGE. Moreover, the allergen‐specific differences observed (most notably reduced lysozyme in certain powdered products) were corroborated by both SDS‐PAGE and MS. These results confirm that the findings reflect genuine differences in allergen composition between commercial EW products rather than methodological bias.

A limitation of this study is that only a single batch of each commercial egg white product was analysed. The use of a single batch was intentional to minimise batch‐related variation and enable direct comparison of allergen composition between products. Furthermore, commercial allergen‐containing products are typically manufactured under quality control processes designed to maximise batch‐to‐batch consistency, including standardisation of source materials and the use of reference preparations for product verification [8]. However, when oral immunotherapy is performed using commercial products, it is recommended to use a single product batch throughout treatment or to assess the allergen content of each batch to ensure consistency [9].

In summary, we identified significant variability in allergen content between powdered and liquid products. Even within the same formulation type, allergen levels differed, with powdered forms showing the greatest inconsistency, although measurable differences were also evident between liquid products. Variability in allergen composition between commercial egg products may contribute to differences in allergen exposure, efficacy and avoidable adverse allergic reactions during oral immunotherapy, particularly if products are interchanged during treatment [9]. Notably, in the present study, ovomucoid (Gal d 1) and ovotransferrin (Gal d 3) appeared relatively resistant to processing‐associated degradation. In contrast, lysozyme (Gal d 4) was markedly reduced in several powdered products, suggesting differential stability of individual allergens during manufacturing. Consequently, products administered at equivalent protein doses may deliver different quantities and proportions of clinically relevant allergens, potentially affecting allergen potency, dose equivalence, and treatment consistency. Although clinical outcomes were not assessed in this study, these findings highlight the importance of allergen characterisation and product standardisation for immunotherapy applications.

## Author Contributions

E.B.J.: Data Curation; Formal Analysis; Investigation; Methodology; Visualisation; Writing – Original Draft Preparation; Review and Editing. S.K.: Data Curation; Formal Analysis; Visualisation; Writing – Review and Editing. E.B.: Investigation; Data Curation; Writing – Review and Editing. P.L.: Conceptualisation; Writing – Review and Editing. A.C.L.: Conceptualisation; Writing – Review and Editing. L.S.‐Y.W.: Conceptualisation; Writing – Review and Editing. A.S.‐Y.L.: Conceptualisation; Writing – Review and Editing. E.H.T.: Conceptualisation; Writing – Review and Editing. L.P.‐C.S.: Conceptualisation; Writing – Review and Editing. M.L.K.T.: Conceptualisation; Funding Acquisition; Methodology; Project Administration; Validation; Writing – Review and Editing. A.L.L.: Conceptualisation; Funding Acquisition; Methodology; Supervision; Validation; Writing – Original Draft Preparation; Writing – Review and Editing. All the authors were involved in the final approval of the article.

## Funding

This study was funded by a National Health and Medical Research Council (NHMRC) grant—Australia, Project Grant—PEAT Study. The Murdoch Children's Research Institute is the sponsor of the study. The study was conducted at James Cook University.

## Ethics Statement

Ethics approval was obtained from the Human Research Ethics Committees at the Royal Children's Hospital (HREC 2019.082) and National Healthcare Group Domain Specific Review Board (NHG DSRB) (2019/00029) to utilise patient sera in this study.

## Consent

Written informed consent was obtained for all study participants from their parents or guardians. Consent was voluntary and free from coercion, and participants were free to withdraw at any time without this affecting their future care. The confidentiality of participants was protected at all times.

## Conflicts of Interest

M.L.K.T. is a past member of Nestle Nutrition Institute Medical Advisory Board Oceania, past member of Nutricia global scientific advisory board; received speaker fees from Nestle Nutrition Institute and Abbott Nutrition; consultant to Bayer Pharmaceuticals; received research funding from Abbott Nutrition, Bayer Pharmaceuticals, Prota Therapeutics; employee of Prota Therapeutics and inventor on a patent owned by MCRI “A method for inducing tolerance”. L.P.‐C.S. has received reimbursement for speaking at conferences sponsored by Danone and Nestle and consulting for Mead Johnson and Nestle. L.P.‐C.S. has received research funding from Danone. A.L.L. has received funding from Aimmune Therapeutics.

## Data Availability

The data (patient data table, patient immunoblots, and egg white extracts visualised by SDS‐PAGE) that support the findings of this study are openly available in Zenodo at https://zenodo.org/records/19674183.
